# Screening and hospitalization of breast and cervical cancer in Brazil from 2010 to 2022: A time-series study

**DOI:** 10.1371/journal.pone.0278011

**Published:** 2023-10-26

**Authors:** Haryelle Náryma Confessor Ferreira, Gustavo Nepomuceno Capistrano, Thiffany Nayara Bento de Morais, Ketyllem Tayanne da Silva Costa, Ana Luiza Santos Quirino, Roberta Letícia Pimentel da Costa, Fábia Barbosa de Andrade

**Affiliations:** 1 Department of Public Health, Master in Public Health, Federal University of Rio Grande do Norte, Rio Grande do Norte, Brazil; 2 Department of Nursing, Federal University of Rio Grande do Norte, Rio Grande do Norte, Brazil; 3 Doctor of Heath Science, Department of Nursing, Federal University of Rio Grande do Norte, Rio Grande do Norte, Brazil; Instituto Butantan, BRAZIL

## Abstract

In Brazil, during the pandemic caused by COVID-19, screening for breast and cervical cancers was postponed or interrupted due to the prevailing health conditions. These neoplasms, however, are responsible for high morbidity and mortality among women in Brazil and have a major impact on the quality of life of this population and public health. Thus, this study aims to evaluate the epidemiological behavior of hospitalization for cervical and breast cancer in Brazilian women, as well as the trend of screening tests of breast and cervical cancer in the years 2010 to 2022 according to the regions of Brazil. This is ecological research of time series, based on secondary data obtained from information systems of the country, about hospital admissions for breast and cervical cancer and screening methods used for these tumors. The data were analyzed in the Joinpoint Regression Program, to obtain the linear regression and temporal analysis of the variables. As a result, between 2010 and 2022, the rates of mammography varied from 36 to 71 exams, while in the pap smear, the variations were 126 and 226 cytopathological exams per 1000 women. Hospitalizations for these cancers peaked in 2019, with 48 hospitalizations for cervical cancer per 100,000 women and 147 hospitalizations for breast cancer for the same population. For both, in the pandemic years, between 2020 and 2022, there is a decrease in Brazil and in all its regions. As for the tracking of these diseases, it was observed that the performance of mammograms and preventive tests showed a similar behavior, in which there is a higher supply of these tests until 2019 and a drop during the pandemic period. This leads to the conclusion that even though Brazil has several policies for the screening of these diseases, there is still instability in the offering of these tests and that there was instability in this area during the pandemic.

## Introduction

The discrepancy in cancer occurrence across different regions of the world can be primarily attributed to a diverse range of socioeconomic, environmental, and behavioral factors. Developing countries, in particular, often grapple with limited resources that hinder their capacity to effectively detect early-stage cancer cases. Unlike their counterparts in developed nations, where screening programs such as mammograms, prostate-specific antigen tests, colonoscopies, and Pap smears are commonplace, individuals in developing countries are frequently deprived of comprehensive screening initiatives [1].

Also, it is widely believed that the majority of cancer cases (over 90%) arise from the intricate interplay between genetic variations, environmental influences, and lifestyle choices, that said, besides genetic factors also exert influence, their predominant impact is confined to a relatively small segment of the population [1].

Furthermore, Cervical Cancer (CC) is in the fourth position of the most frequent tumors among women worldwide [2]. This cancer represents a considerable indicator of inequality since approximately 85% of its cases affect women with low education and low socioeconomic conditions [3].

The SARS-CoV-2 virus, responsible for the COVID-19 pandemic, has negatively impacted elective care in most countries. This includes cancer screening, as these services have been interrupted to prioritize the reduction of the risk of spreading the new coronavirus in health services [4].

Therefore, because it is a public health problem with a global impact, high mortality rates, and a direct influence on the quality of life of the patient, it is important to encourage studies on this topic to try to understand the impacts that the pandemic had on early detection and screening of cancer.

Thus, this study aims to evaluate the epidemiological behavior of hospital admissions for cervical and breast cancer in Brazilian women, as well as the performance of screening tests for these diseases in the years from 2010 to 2022 in Brazil.

## Materials and methods

The present study is ecological time-series research, carried out using data obtained from secondary sources. It is a study that took place in Brazil and its main focus is the epidemiological behavior of screening of breast and cervical cancer between 2010 and 2022. The main reason for the analysis of the last twelve years of hospitalizations for these neoplasms and their screenings is to identify how these variables have behaved and if the COVID-19 pandemic influenced screening and early diagnosis.

### Data collection

The data were collected on October 26, 2022 and updated on June 05, 2023 from the national platform of Brazilian public health data called DATASUS, and the data for the present study were taken from four information systems: 1) Hospital Information System (SIH); 2) Cervical Cancer Information System (SISCOLO); 3) Breast Cancer Information Number of hospitalizations and exams offered to the population in Brazilian Primary Care. For the analysis of the profile of cytopathological exams, mammograms and hospitalizations, data from the period 2013 to 2022 were used. The data can be consulted on the site <https://datasus.saude.gov.br/informacoes-de-saude-tabnet/>.

### Study variables

The dependent variables of the study are: hospitalizations for cervical and breast cancer; the number of cytopathological exams of the uterus in the Brazilian screening age group of 25 to 69 years; the number of mammograms performed in the age group between 50 and 69 years. This variable corresponds to the number of tests performed and not the number of women screened. The independent variable used was the period of time from January 1, 2010 to December 31, 2022, and Brazilian regions.

### Information analysis

To achieve the objective of the study, an analysis was performed from the raw data, calculating the rate of hospitalization for cervical and breast cancer, separately, according to Chapter II of ICD-10 entitled "neoplasms (tumors)", where the data obtained for breast cancer concerns only the malignant tumors of code C50 of the ICD-10 morbidity list, while for cervical cancer the corresponding code is C53, also referring only to malignant tumors, in addition to the rates of performance of cervical cytopathological exams and mammography by regions of Brazil. In order to do this, it was necessary to divide the number of hospitalizations (H) and exams by the number of the analyzed population, which are women of all ages to calculate the overall hospitalization, women in the age group 25 to 69 years for the cytopathological exam, and between 50 and 69 years for the mammography denominator, and finally multiplied by 100,000 for hospitalizations and multiplied by 1,000 in the case of exams, as shown in the equations below.

$$\text{H}=\left(\text{Hospitalization}\;\text{for}\;\text{breast}\;\text{cancer}/\text{women}\;\text{of}\;\text{all}\;\text{ages}\right)\;\text{X}\;100,000$$ (1)

$$\text{H}=\left(\text{Hospitalization}\;\text{for}\;\text{cervical}\;\text{cancer}/\text{women}\;\text{of}\;\text{all}\;\text{ages}\right)\;\text{X}\;100,000$$ (2)

$$\text{H}=\left(\text{Mammograms}/\text{women}\;\text{aged}\;25–69\right)\;\text{X}\;1,000$$ (3)

$$\text{H}=\left(\text{Cervical}\;\text{cytopathology}/\text{women}\;\text{aged}\;50–69\right)\;\text{X}\;1,000$$ (4)

Thus, Eqs 1 and 2 are associated with gross hospitalizations for breast and cervical cancer, so Eq 1 was performed by dividing the number of hospitalizations for breast cancer, divided by the number of women of all ages and then multiplied by 100,000. In calculation 2, the division was idealized through the quantification of hospitalization for cervical cancer and divided by the number of women of all ages, and the result was multiplied by 100,000. With regard to Eqs 3 and 4, they are linked to the control tests for these types of cancer, so Eq 3 was divided by the number of mammograms divided by the number of women aged 25 to 69 years old and multiplied by 100 thousand, finally, Eq 4 contains the division number of cytopathological examinations of the uterine cervix by the number of women who are 50 to 69 years old and subsequently multiplied by 100 thousand.

After being collected, the data downloaded in CSV were processed and stored in Microsoft Excel^®^, where the database treatment occurred, selecting the important data for the research. The database was formatted to the standards of the JoinPoint^®^ program, a software used in the statistical analysis of cancer morbidity and mortality, as it allows trends to be observed, whether flat, ascending, or descending, using Joinpoint models, guiding a historical series through Poisson regression to estimate Annual Percentage Change (APC) and Average Annual Percentage Change (AAPC) that can be consulted on the site <https://surveillance.cancer.gov/>. For each trend detected, a 5% significance level was used [5]. Joinpoint is an accessible and free software that allows significant statistical analysis. However, it is only possible to correlate up to two variables.

### Ethical support

The information obtained for the elaboration of the research came from secondary sources and was collected by public domain databases and, therefore, did not need to be submitted to the Research Ethics Committee appreciation, as recommended by the Brazilian Resolution No. 510, of April 07, 2016 [6]. No declaration of consent required.

## Results

**Fig 1:**
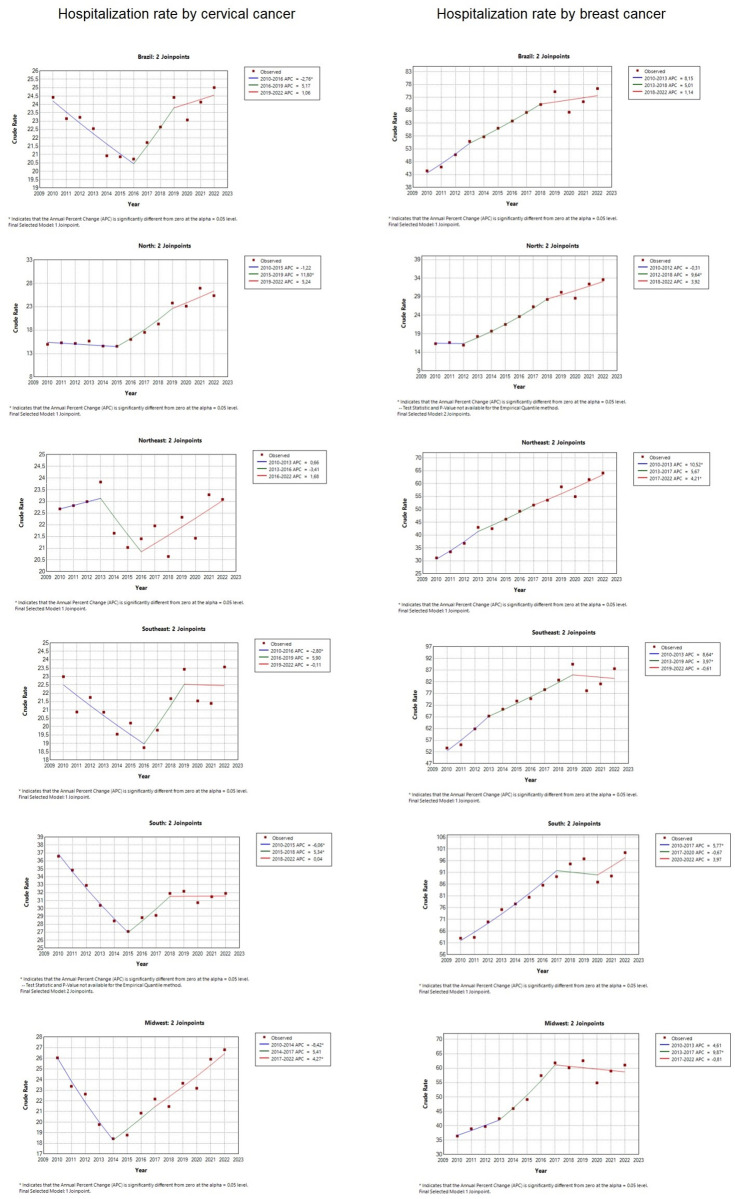
Hospitalization rate for cervical and breast cancer in Brazil, 2010 to 2022. Brazil, 2022.

The results obtained can be seen in Fig 1, which shows the rates of hospitalization for cervical and breast cancer. In Brazil, it is possible to observe a downward behavior in hospitalizations for cervical neoplasia between the years 2010 and 2015, which went from 47 hospitalizations per 100,000 women to 41 hospitalizations per 100,000 women. Then, it showed an increase until 2019, when it peaked with more than 48 hospitalizations per 100,000 women.

The North region shows a more accentuated growth between 2015 and 2021, increasing from 33 to 58 hospitalizations per 100,000 women. The Northeast region shows more stability in hospitalization rates, varying between 43 and 50 hospitalizations per 100,000 women, with the except for 2022, which showed 35 hospitalizations per 100,000 women. The Southeastern, Southern, and Midwestern regions show a graph similar to Brazil, but with special emphasis on the South, which shows the highest hospitalization rates, ranging from 51 to 69 hospitalizations per 100,000 women, and reaching the lowest number in 2022, with 42 hospitalizations per 100,000 women.

In the pandemic years, between 2020 and 2022, the graph shows a downward trend in Brazil and in all regions, showing a reduction in hospitalizations.

Table 1, which presents the statistical data of the linear regression of cervical cancer hospitalization in Brazil and their respective regions between 2010 and 2022, shows the presence of two Joinpoints in Brazil, in 2015 and 2020, the same occurs in the Northern, Southeastern and Southern regions. The behavior changes in the Northeast regions, where the first Joinpoint is later, in 2017, and in the Midwest, which presents the earliest first Joinpoint, in 2014.

**Table 1:** Joinpoint analysis of cervical cancer hospitalization rate in Brazil and its regions, 2010 to 2022. Brazil, 2022.

Local	Joinpoint^1^	Period	APC^2^	Lower	Upper	AAPC^3^	Lower	Upper
Brazil	2010, 2016, 2019	2010 to 2016	-2.7*	-3.8	-1.6	0.1	-1.3	1.5
		2016 to 2019	5.1	-1.4	12.2			
		2019 to 2022	1.0	-2.1	4.4			
North	2010,2015, 2019	2010 to 2015	-1.2	-4.7	2.4	4.5	1.6	7.6
		2015 to 2019	11.6	2.9	21.4			
		2019 to 2022	5.2	-3.0	14.2			
Northeast	2010, 2013, 2016	2010 to 2013	0.6	-5.3	7.0	0.1	-2.5	2.9
		2013 to 2016	-3.4	-14.5	9.2			
		2016 to 2022	1.6	-0.4	3.8			
Southeast	2010, 2016, 2019	2010 to 2016	-2.8*	-4.9	-0.5	-0.0	-2.9	3.0
		2016 to 2019	5.9	-7.3	21.0			
		2019 to 2022	-0.1	-6.5	6.8			
South	2010, 2015, 2018	2010 to 2015	-6.0*	-7.4	-5.1	-1.2*	-1.6	-0.9
		2015 to 2018	5.3	3.1	6.8			
		2018 to 2022	0.0	-2.2	1.0			
Midwest	2010, 2014, 2017	2010 to 2014	-8.4*	-11.6	-5.0	0.1	-2.3	2.6
		2014 to 2017	5.4	-5.9	18.1			
		2017 to 2022	4.2	1.6	6.9			

^1^annual percentage change.

^2^average annual percentage change.

*p-value <0.05.

On the other hand, the hospitalization rates for breast cancer in Brazil and regions exceed the numbers for cervical cancer. In the national context, breast cancer shows a gradual growth behavior until 2019, peaking at 147 hospitalizations per 100,000 women. Southeast and South had higher peaks than Brazil, especially the latter, with 181 hospitalizations per 100,000 women. All regions grew to a peak in 2019 and then began to significantly regress the hospitalization rate, which shows a downward trend during the pandemic.

Table 2, which presents the statistical data of the linear regression of breast cancer hospitalization in Brazil and their respective regions between 2010 and 2022, shows the presence of two Joinpoints in Brazil, in 2013 and 2019, the same occurs in the Southeast and South regions. On the other hand, in the North region, a regression of Joinpoint beginning in 2012 is perceived, the shortest observed. Being, therefore, a scenario similar to that of cervical cancer. The behavior changes in the Northeast, where the second Joinpoint is later, in 2020, and in the Midwest, where the first Joinpoint is later, in 2016.

**Table 2:** Joinpoint analysis of breast cancer hospitalization rate in Brazil and its regions, 2010 to 2022. Brazil, 2022.

Local	Joinpoint^1^	Period	APC^2^	Lower	Upper	AAPC^3^	Lower	Upper
Brazil	2010,2013, 2018	2010 to 2013	8.1	-0.4	17.4	4.4*	1.7	7.2
		2013 to 2018	5.0	-0.3	10.6			
		2018 to 2022	1.1	-4.0	6.5			
North	2010, 2012, 2018	2010 to 2012	-0.3	-3.9	5.6	6.0*	5.2	6.8
		2012 to 2018	9.6*	8.4	13.6			
		2018 to 2022	3.9	-0.0	5.9			
Northeast	2010, 2013, 2017	2010 to 2013	10.5*	3.5	17.9	6.2*	3.8	8.5
		2013 to 2017	5.6	-0.9	12.7			
		2017 to 2022	4.2*	1.2	7.2			
Southeast	2010, 2013, 2019	2010 to 2013	8.6*	4.6	12.7	3.9*	2.7	5.1
		2013 to 2019	3.9*	2.2	5.7			
		2019 to 2022	-0.6	-4.2	3.1			
South	2010, 2017, 2020	2010 to 2017	5.7*	4.6	6.9	3.8*	1.8	5.8
		2017 to 2020	0.6	-8.4	7.8			
		2020 to 2022	3.9	-4.2	12.8			
Midwest	2010, 2013, 2017	2010 to 2013	4.6	-4.1	14.2	4.0*	0.8	7.2
		2013 to 2017	9.8*	0.6	19.9			
		2017 to 2022	-0.8	-4.6	3.1			

^1^annual percentage change.

^2^average annual percentage change.

*p-value <0.05.

**Fig 2:**
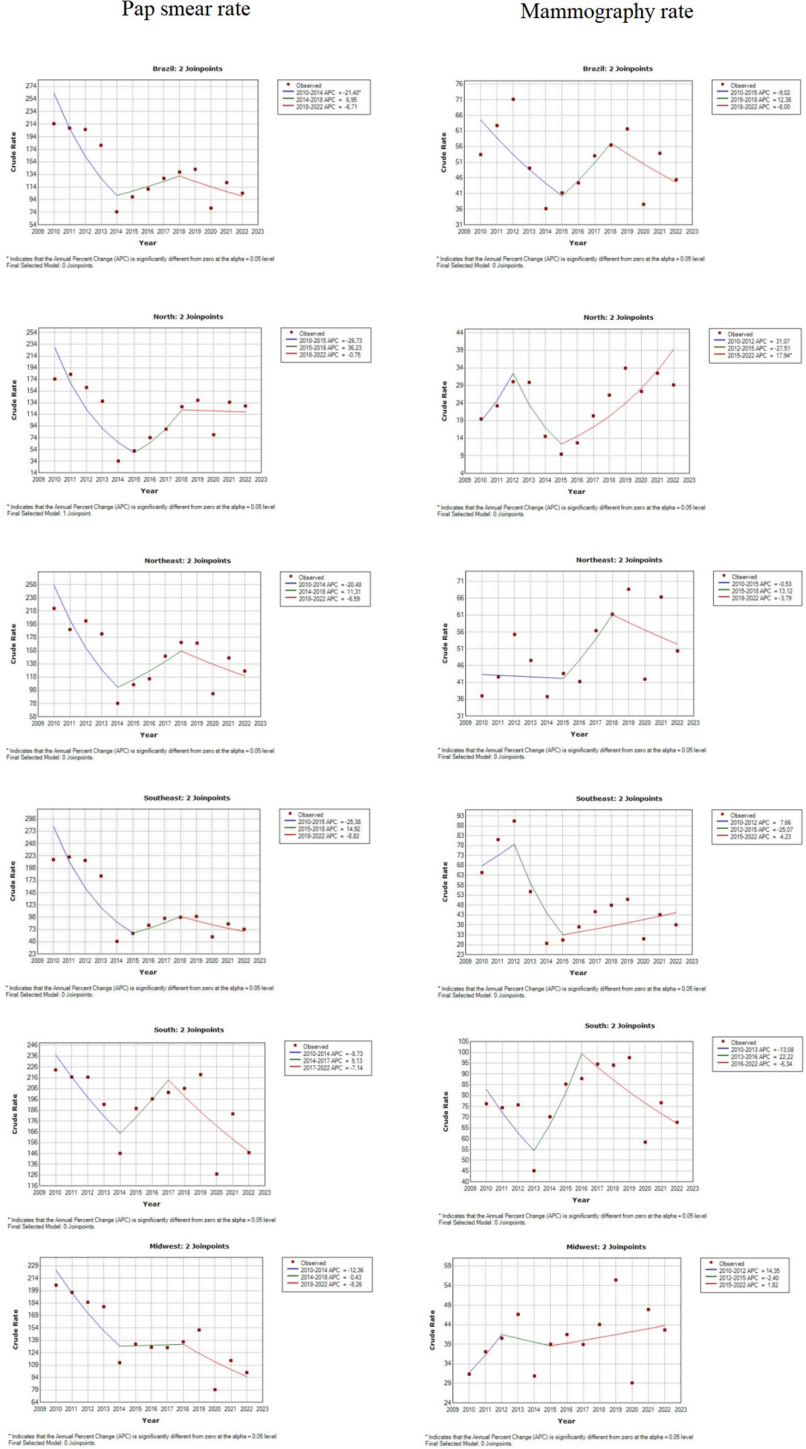
Pap smear and mammography rate in Brazil, 2010 to 2022. Brazil, 2022.

In Fig 2, it is shown the rate of cervical cytopathological exams and mammography performed from 2010 to 2022 in all regions of Brazil. Thus, it was possible to observe that in all charts of cytopathological tests of the uterine cervix, the behavior of the curve first goes through a drop, then the number of tests performed rises, and, finally, in the year 2020, there was a significant decrease in the number of these exams, a period that coincides with the beginning of the pandemic.

In Brazil, the rates of mammography vary between 36 and 71 exams per 1000 women, with special emphasis on the year 2014, which represents the worst index of the analyzed period, although there is growth afterward, the numbers fall again starting in 2020. The same behavior is repeated in all regions of the country. The South is the region with rates that vary in a higher range, between 126 and 226 cytopathological exams per 1000 women. On the other hand, the North region presents the lowest statistics, between 34 and 182 exams per 1000 women.

Table 3, which presents the statistical data of the linear regression of the performance of cervical cytopathological exams in Brazil and their respective regions, between 2010 and 2022, shows the presence of two Joinpoint in Brazil, in 2014 and 2018, the same occurs in the Northeast and Midwest regions, differently from what was observed in hospitalizations. The behavior changes in the North and Southeast regions, where the first Joinpoint is later, in 2015, and in the South, which has the second earliest Joinpoint, in 2017.

**Table 3:** Joinpoint analysis of Pap smear rate in Brazil and its regions, 2010 to 2022. Brazil, 2022.

Local	Joinpoint^1^	Period	APC^2^	Lower	Upper	AAPC^3^	Lower	Upper
Brazil	2010, 2014, 2018	2010 to 2014	-21.4*	-36.2	-3.1	-7.8	-17.6	3.2
		2014 to 2018	7.0	-23.1	48.8			
		2018 to 2022	-6.7	-24.3	15.0			
North	2010, 2015, 2018	2010 to 2015	-26.7	-48.5	4.2	-5.3	-32.9	33.6
		2015 to 2018	36.2	-71.8	558.2			
		2018 to 2022	-0.8	-39.7	63.3			
Northeast	2010, 2014, 2018	2010 to 2014	-20.5	-36.9	0.2	-6.1	-17.2	6.3
		2014 to 2018	11.3	-22.8	60.5			
		2018 to 2022	-6.6	-25.9	17.7			
Southeast	2010, 2015, 2018	2010 to 2015	-25.4	-46.5	4.1	-11.1	-35.8	23.1
		2015 to 2018	14.9	-74.1	410.0			
		2018 to 2022	-8.8	-43.1	46.1			
South	2010, 2014, 2017	2010 to 2014	-8.7	-24.8	10.7	-3.9	-15.9	9.9
		2014 to 2017	9.1	-40.7	100.9			
		2017 to 2022	-7.1	-19.0	6.4			
Midwest	2010, 2014, 2018	2010 to 2014	-12.4	-29.6	9.1	-6.9	-17.2	4.8
		2014 to 2018	0.4	-28.9	41.9			
		2018 to 2022	-8.3	-26.3	14.2			

^1^annual percentage change.

^2^average annual percentage change.

*p-value <0.05.

Regarding mammography exams, a unique behavior is observed in each region of the country. Nationally, the rates vary between 36 and 71 mammograms per 1000 women, with 2014 being the year with the lowest rate. However, it shows growth until 2019, falling again in 2020 and the following years, as do the Southeast and Midwest. The North, however, manages to increase the number of mammograms starting in 2015 and stabilize the number of the exams during the pandemic years. The Northeastern and Southern regions show an upward trend, peaking in 2019, with 69 and 98 mammograms per 1000 women, respectively, but reduce the amount offered during the pandemic.

Furthermore, it is noteworthy that the Southern region stood out in the number of mammograms offered in the studied period, which varied between 45 and 98 exams per 1000 women. The North region, on the other hand, is a negative highlight in this aspect, since it presented rates that ranged between 9 and 34 mammograms per 1000 women.

Table 4, which presents the statistical data of the linear regression of the performance of mammography in Brazil and their respective regions between 2010 and 2022, shows the presence of two Joinpoints in Brazil, in 2015 and 2018, the same occurring in the Northeast regions. The behavior changes in the Northern, Southeastern and Midwestern regions, where the Joinpoint were earlier, being in 2012 and 2015, and in the South, which also presents earlier Joinpoints, in 2013 and 2016.

**Table 4:** Joinpoint analysis of mammography rate in Brazil and its regions, 2010 to 2022. Brazil, 2022.

Local	Joinpoint^1^	Period	APC^2^	Lower	Upper	AAPC^3^	Lower	Upper
Brazil	2010, 2015, 2018	2010 to 2015	-9.0	-25.1	10.4	-3.0	-19.8	17.2
		2015 to 2018	12.4	-52.8	167.5			
		2018 to 2022	-6.0	-28.5	23.7			
North	2010, 2012, 2015	2010 to 2012	31.1	-43.5	204.1	6.3	-12.9	29.7
		2012 to 2015	-27.5	-68.8	68.2			
		2015 to 2022	17.9*	5.4	32.0			
Northeast	2010, 2015, 2018	2010 to 2015	-0.5	-17.7	20.2	1.6	-15.6	22.3
		2015 to 2018	13.1	-51.6	164.2			
		2018 to 2022	-3.8	-26.4	25.8			
Southeast	2010, 2012, 2015	2010 to 2012	7.7	-44.8	110.1	-3.5	-17.6	13.0
		2012 to 2015	-25.1	-61.6	46.2			
		2015 to 2022	4.2	-4.7	14.0			
South	2010, 2013, 2016	2010 to 2013	-13.1	-33.7	13.9	-1.7	-12.9	10.8
		2013 to 2016	22.2	-28.8	109.9			
		2016 to 2022	-6.3	-14.5	2.6			
Midwest	2010, 2012, 2015	2010 to 2012	14.3	-48.1	151.8	2.7	-14.8	23.8
		2012 to 2015	-2.4	-55.7	114.9			
		2015 to 2022	1.8	-8.4	13.1			

^1^annual percentage change.

^2^average annual percentage change.

*p-value <0.05.

Table 5 refers to the epidemiological profile analyzed exams and hospitalizations that occurred in the period from 2013 to 2022, showing a higher prevalence of cytopathological exams in women aged 25 to 39 years old, who have an elementary school education and an increase in hospitalization for cervical cancer as of the year 2018. In regards to the mammography data, it was possible to analyze that there is a higher prevalence in women aged 50 to 60 years of age, with an elementary school education and there was an increase in hospitalization for Breast cancer from 2017.

**Table 5:** Profile of cervical cytopathological exams, mammography and hospitalization for cervical and breast cancer, 2013 to 2022. Brazil, 2022.

YEAR			2013	2014	2015	2016	2017	2018	2019	2020	2021	2022
**Pap Smears**	**Age (%)**	**25 to 39**	41,30	41,66	41,14	40,81	40,50	40,07	39,54	38,79	37,71	36,99
		**40 to 54**	38,38	38,42	38,52	38,50	38,65	38,78	38,85	39,57	39,99	39,64
		**55 to 69**	20,32	19,92	20,34	20,69	20,85	21,15	21,60	21,63	22,29	23,37
	**Education (%)**	**Illiterate**	4,31	4,46	4,26	5,47	8,28	7,78	4,43	2,91	5,26	0,00
		**Elementary School**	62,75	60,48	58,81	65,43	69,75	62,78	49,57	49,51	68,42	25,00
		**High school**	28,18	29,2	30,53	24,80	18,15	25,56	39,01	41,75	23,68	25,00
		**University education**	4,76	5,86	6,40	4,30	3,82	3,89	6,98	5,83	2,63	50,00
**Hospitalization**	**Cervical Cancer (%)**		9,98	9,25	9,23	9,17	9,61	10,02	10,80	10,20	10,68	11,06
**Mammographies**	**Age (%)**	**50 to 60**	64,86	24,38	63,86	63,49	62,79	62,13	61,8	62,08	61,93	60,31
		**60 a 69**	35,14	89,68	36,14	36,51	37,21	37,87	38,2	37,92	38,07	39,69
	**Education (%)**	**Illiterate**	5,25	7,22	6,11	7,12	6,02	5,36	3,33	0	0	0
		**Elementary School**	62,36	67,73	70,2	70,6	69,17	61,2	58,33	66,67	0	100
		**High school**	28,44	20,61	19,33	19,66	20,8	27,13	32,08	33,33	0	0
		**University education**	3,94	4,45	4,36	2,62	4,01	6,31	6,25	0	0	0
**Hospitalization**	**Breast Cancer (%)**		8,39	8,66	9,16	9,58	10,08	10,55	11,30	10,09	10,71	11,48

## Discussion

Screening and early diagnosis are characterized as two strategies for the detection of cancer, which is a pathology that generates a high morbidity rate in Brazil, being a public health problem, especially when it affects priority groups, such as women, who have a high incidence of cervical and breast cancer, as shown in Fig 1, Tables 1 and 2. Early diagnosis allows higher chances of cure, better prognosis, and lower risk of hospitalization and reduces hospitalization costs [7].

Cervical Cancer (CC) is the third most prevalent type of cancer among women in Brazil, with HPV (Human Papilloma Virus) as the main cause [8]. Despite the high prevalence, the screening services offered by Unified Health System (SUS), despite being opportune, show a decrease in the incidence of this type of cancer, as well as the mortality caused by it [9].

The transition to fully effective primary HPV screening can be time-consuming and complicated. The Netherlands, for example, was the first country in Europe to implement primary HPV screening in 2017. The new program started only after a preparatory phase that lasted more than 4 years [10].

For the diagnosis and screening of CC, in Brazil, the main test used in SUS is the Pap smear or cytopathological examination of the uterine cervix, guaranteed to all people with a uterus, in the age group of 25 to 64 years, who have already started sexual life, throughout the national territory. This age group is a priority because it has a higher incidence of high-grade lesions that are precursors of cervical cancer [11].

However, there are still difficulties in making this procedure fully available, as an example, there is the exit, in 2008, of the Pap smear from the Fund for Strategic Actions and Compensation (FAEC), which is a public budget intended exclusively for strategic actions. In this funding model, the procedure was performed and registered in the system for direct receipt of funds [12].

Since 2008, Pap smears have been funded by the Medium and High Complexity Funding (MAC), which transfers the financial resources to state and municipal managers to distribute among all the procedures performed in the service [13]. This action may have contributed significantly to the reduction in the performance of cervical cytopathological exams nationwide between 2010 and 2014, as shown in Fig 2, Tables 3 and 4.

Another difficulty encountered is the periodicity in which the Pap smear is performed since the Ministry of Health recommends that it should be done every three years when the woman has a healthy cervix. However, data show that only 8% of the exams are performed every three years, while there are about 50% of annual repetitions. These data show that some women do not perform the exam regularly, while others repeat it excessively [13].

According to Fig 2, it is possible to observe that from 2014 on, there is an increase in the rate of cervical cytopathological exams, an occurrence that may be linked to the new Brazilian norm through Ordinance no. 3.388, of December 30, 2013, which redefines the National Qualification in Cytopathology in the prevention of cervical cancer (QualiCito), aiming to increase the coverage of cytopathological exams, collect samples with a higher level of quality, devise means of improving professionals, in addition to applying incisive monitoring through SISCAN [14].

Breast carcinoma is a heterogeneous disease and comprehends biologically distinct neoplasms, with varied clinical and morphological symptoms [12]. Thus, mammography is the gold standard test for early diagnosis and screening of this neoplasm. The recommended age range for mammography is 50 to 69 years, once every two years [12].

This orientation is implemented in most countries that have adopted organized screening for breast neoplasia, due to evidence proving an effective impact on mortality reduction and improved risk-benefit, which is not noted in other age groups [15,16].

However, it is observed a divergence of situations related to the provision of mammography equipment in Brazil, covering several adversities in the organization of the service, such as scarcity of available equipment, trained professionals, obstacles of access related to geographic distance, and underutilization of equipment [17].

Thus, various barriers, such as socioeconomic challenges and limited healthcare access, hinder adherence to preventive practices. Research reveals that sociodemographic factors like employment, education, and income affect access to healthcare services and significantly influence adherence to mammography and Pap smear screening (Table 5). Lower educational levels and income are particularly associated with greater difficulties in accessing healthcare for this population [18,19].

This problem is reflected in the results of the present study (Fig 2), in such a way that the South and Southeastern regions, with greater health structures and greater financial resources, present higher rates of mammography, demonstrating the heterogeneity in public health in the country.

Worldwide, breast cancer is the most common cancer among women. The highest incidence rates found in studies were in Australia and New Zealand, the countries of Northern Europe and Western Europe. Research shows that regardless of the socioeconomic factors in the country, new cases of breast cancer are in the top positions of malignant neoplasms in women [20,21].

Nevertheless, in developed countries, a decrease in the incidence of breast neoplasms has been detected, which may be associated with the decrease in hormone replacement treatment in women in the climacteric period [20,21], but this behavior is different from what is observed in Brazil, because, as shown in Fig 1, between 2010 and 2019, hospitalizations for breast cancer follow a gradual increase in hospitalization.

A factor of great impact in recent years has been the pandemic caused by COVID-19, which has reflected on the performance of examinations and surgical procedures worldwide [22]. The results of the authors Demarchi et al. [23], who analyzed a decrease of 1,705,475 mammograms in Brazil in the year 2020 alone, show a reduction of about 40% in relation to the previous year.

Nonetheless, according to the results obtained in this study, some regions, such as the North, Midwest, and Southeast, showed an upward behavior in the performance of mammograms. This finding may be related to the increase in financial resources received by the states and municipalities to invest in the health system during the pandemic period [24].

Thus, the Society of Breast Imaging proposed delaying mammograms by “several weeks or a few months” due to quarantine [25]. In contrast, the Canadian Society of Breast Imaging and the Canadian Association of Radiologists proposed that all mammograms should be delayed for at least 6 to 8 weeks [26]. However, in the country, the Brazilian Society of Mastology (SBM) issued in March a statement attesting that the conduct should be determined according to the local reality, taking into consideration the risks and demands posed by the pandemic [27], which corroborates the different rates of mammography among the regions, as demonstrated in Fig 2.

During the early months of the pandemic, there was a significant decrease in the number of Pap smears performed in Ontario, Canada. In the worst month, there was an 85.8% drop, and overall, there was a 63.8% decline between March and August 2020 [28].

Another study, conducted in the United States, California, points to a 78% reduction in the monthly rate of Pap smears performed by women aged 21 to 29 years and 82% in women aged 30 to 65 years [29]. This pattern is not very different from that observed in the present study, in which Brazil, showed a drop from 134 preventive exams per thousand women to approximately 95 exams from 2018 to 2022, respectively, reflecting the impact of the pandemic period on the performance of these exams.

Therefore, the limitations of this study were related to the availability of data regarding the quantification of materials made available for the performance of mammography and Pap smears nationwide, the absence of data regarding the educational profile of women who had mammograms in some years of the study, as well as more precise information regarding the financing of these materials. In addition, there is a fragmentation of data in the Health Information Systems regarding hospitalizations for breast and cervical cancer and the number of tests performed for each woman in the country.

## Conclusion

Knowing that breast cancer and cervical cancer occupy significant positions in female morbidity and mortality in Brazil, it is prudent to point out suitable solutions to combat these diseases. Thus, the screening methods must be a priority, with the standardization of the wide attendance and availability of Pap smears and mammograms throughout the country.

However, currently, there are impasses for the full offer of these procedures, even with the various public policies for the promotion of this field. In addition, it was observed in the study that hospitalization rates for these neoplasms had a gradual increase before the pandemic and that, after 2020, there was a reduction both in hospitalization and in the supply of diagnostic tests. Therefore, there is a need for further studies that highlight the impact of the pandemic period on the detection, screening, and early diagnosis of these diseases.
